# Rush Medical College

**Published:** 1874-12

**Authors:** Walter Hay


### Clinics.

***Rush Medical College.*** Diseases of Brain and Nervous System, by Dr. Walter Hay. Reported by Henry L. Harrington.

*Case of Progressive Locomotor Ataxia.*

Man, aged 45; laborer. States that he has been in the habit of working along the docks, and has been much exposed to cold and wet. Was well until about one year ago, when he began to have pricking and tingling sensations of the legs and feet, also sharp darting pains in the same limbs. He soon found it difficult to walk; could not control his movements; could not walk well in the dark; was unable to stoop over and wash his face without falling; felt all the time as if he was standing on pincushions; he has continued growing worse until the present time.

On examination, find patient well nourished; appetite good; bowels constipated; does not urinate easily; sleeps well. Patient is unable to stand with his eyes shut, and his feet close together; in walking extends his legs powerfully, but irregularly, and brings the foot down with a slapping motion, the heel striking first; keeps his eyes fixed constantly upon his feet when walking; all his motions are unsteady and irregular; when sitting down he can move his legs with ease in all directions; has lost no muscular power. There is anæsthesia of the soles of his feet; special sensation unimpaired; intellection good. He was ordered—R. Fluid ext. secale cornut. dr. j, at bedtime.

*Remarks.* This, gentlemen, is a disease which has its seat in the posterior columns of the spinal cord. It is essentially an inflammation of the connective tissue, resulting in hypertrophy and induration of that tissue, and consequent atrophy of true nerve tissue, fibres and cells. The manifestations of the disease are due to the fact that impressions received by the peripheral nerves of sensation are not transmitted to the brain, but are intercepted in the posterior columns at the seat of disease. It is due to this fact that the patient is unable to co-ordinate muscular movements without keeping his eyes constantly fixed upon his limbs. Prognosis is grave; the progress of the disorder may be arrested for a time, but almost certainly returns, and progresses to a fatal issue. Its average duration is from two to five or ten years. The indications for treatment are to diminish the supply of blood to the cord, which is well fulfilled by ergot.

*Two Cases of Chorea.*

CASE I. Girl, aged 10. The mother states that she has suffered from irregular twitchings of the muscles for eighteen months. She has had great difficulty in eating, dropping or spilling whatever she was conveying to the mouth; cannot walk well; falls frequently; is very irritable, crying from slight causes.

On examination, find patient very anæmic and poorly nourished; skin cool; tongue pale; conjunctivæ pearly; appetite poor; bowels costive; sleeps poorly. The child cannot sit still; her hands and feet twitch irregularly; also the muscles of the face and eyes. Ordered—R. Ferri et quiniæ citratis, gr. ij, strychniæ sulph. gr. $\frac{1}{50}$, three times daily.

CASE II. Boy aged 7. Mother states that he was well until three weeks ago, when a window fell upon his hands, frightening him very badly. Ever since, he has been troubled with irregular involuntary motions of both arms and legs; has to be fed, as he drops his food; cannot walk well; appetite good; bowels regular; sleeps well. Patient is quite anæmic, though not emaciated. Ordered—R. Strychniæ sulph. gr. $\frac{1}{50}$, three times daily.

*Remarks.* These are examples of a disease which is due, in these as in most cases, to debility. There is an impoverished condition of the blood, and consequently deficient nutrition of nerve centres. As a result of this, there is an irregular supply of motor power, to which is due the irregularity of muscular contraction. The prognosis is favorable, usually, about three months being required to effect a cure.

The indications are to increase general nutrition. To accomplish this we give good diet, and tonics, such as iron and quinine; also strychnia, which is one of the best nerve tonics we possess. It aids materially in the management of these cases to have the patients practice rhythmical motions, such as would be necessary in jumping the rope, rolling the hoop, crocheting, etc.

RUSH MEDICAL COLLEGE CLINIC. Dr. GUNN. September 12, 1874. Reported by RALPH E. STARKWEATHER, M.D.

*Abscess in the Right Tibia.*

This patient, a little girl of eight years of age, exhibits a rare form of disease, one of interest to the surgeon, namely, abscess of bone. It usually is very insidious in its onset, and not easily traced to any injury. There is, in the first place, great swelling; then pain, very severe and paroxysmal. This pain may be absent for months, and then return to torment the patient for days. Often the pain, more or less acute, will last for a year or longer, frequently being more distressing at night. The abscess may be greatly disproportioned in size and amount to the bone tumor and constitutional suffering; there may be a very small cavity, containing a few drops of pus.

As to the treatment, when there is evidence of pus, the only course is to open the bone and evacuate the pus, by incision or trephine. If there should be reason to suspect a syphilitic taint, you will do wisely to wait and exhibit the potassium iodide.

*Hare Lip.*

In this patient, a puny infant, the soft palate is fissured, but neither the bony arch nor upper lip is fully cleft. In regard to operating, my rule is, when the child is strong and vigorous, and from four to six or eight months of age, to operate. Babies, as a rule, bear the loss of blood with great difficulty. In the case before you, I shall decline to interfere, because of the bad general condition of the baby. The operation should be done, in children, before they fall into bad habits of speech.

OCTOBER 10.

*Amputation of the Right Thigh—Esmarch's Bloodless Method.*

The limb was removed because of extensive caries of the whole of the tibia, the patient, a man fifty-three years of age, having gradually emaciated from the excessive suppuration, continued through several years, till surgical relief became imperative. Dr. Gunn was assisted by Drs. Parkes, Owens, and Starkweather. The elastic bandage was applied very tightly below the point of discharge, but lightly over the wound, in order that no blood loaded with the partly decomposed material, might be crowded into the general circulation. Ether was administered by Dr. Parkes. The operation was by lateral flaps, at the middle of the thigh ; the outer flap was first made, then the one on the left side. A roller quickly applied retracted the soft parts, and the femur was then sawn through.

Length of time of operation, fifty-five seconds. Less than two ounces of blood were lost. Three principal ligatures were made. Four sutures, on either side, were made, allowing the centre of the wound to remain patulous. Dr. Gunn said that this would facilitate the after management of the case. Formerly, the wound used to be closed up, and an attempt made to heal by first intention ; but the failures were too numerous, and inflammation too constant, to warrant this being longer done. During the civil war, most of the cases at Nashville were lost by pyæmia on the second day after amputation, when the wound has been closed up. The practice was changed, and the wound allowed to gap wide open. A T-bandage and water dressings were the only dressing, and then the cases did well.

